# Therapeutic Potential of Adipose Mesenchymal Stem Cells for Synovial Regeneration: from *In-Vitro* Studies to Clinical Applications

**DOI:** 10.1007/s12015-025-10909-5

**Published:** 2025-06-10

**Authors:** Daniel Levinson, Almog Uziel, Victoria Furer, Ari Polachek, Ori Elkayam, Eyal Gur, Yoav Barnea, Inna Solodeev, Smadar Gertel

**Affiliations:** 1 Department of Reconstructive and Aesthetic Surgery, Tel-Aviv Sourasky Medical Center, Tel-Aviv, Israel; 2 Department of Rheumatology, Tel-Aviv Sourasky Medical Center, 6 Weizmann Street, Tel-Aviv, Israel; 3 Gray Faculty of Medical and Health Sciences, Tel-Aviv University, Tel Aviv, Israel

**Keywords:** Adipose-derived mesenchymal stem cells, Extracellular vesicles, Secretome, Regenerative medicine, Clinical application, Tissue regeneration

## Abstract

Synovial joint disorders affect a substantial proportion of the global population, posing a significant challenge to the individual patient and global healthcare systems. Novel therapeutic strategies for resolving cartilage and synovial damage have recently been investigated. Adipose-derived mesenchymal stem cells (AD-MSCs) emerged as a potential cell-based therapy approach due to their accessibility, abundance, low immunogenicity, immunomodulatory effect, and tissue repair capability. The translation of AD-MSCs-based therapies from bench to clinical practice has shown promising results; with extensive evidence supporting their feasibility and efficacy for treating joint disorders. Despite their considerable potential, however, few AD-MSCs-based therapies have been approved for clinical application, primarily due to a lack of standardization and a poorly understood mechanism of action in vivo. The characterization of AD-MSCs from in vitro to in vivo models and eventually to clinical trials enables a comprehensive assessment of their therapeutic potential in synovial regeneration, bridging the gap between basic research and clinical application. The advantages and limitations collected from studies that delineate the effect of AD-MSCs on synovial cells will help researchers translate this cell-therapy approach from bench to clinical application. This review examines current models and applications of their therapeutic potential for synovial regeneration from in vitro studies to clinical trials. We also discuss the potential of cell-free therapy for joint disorders by means of extracellular vesicles.

**Graphical Abstract:**

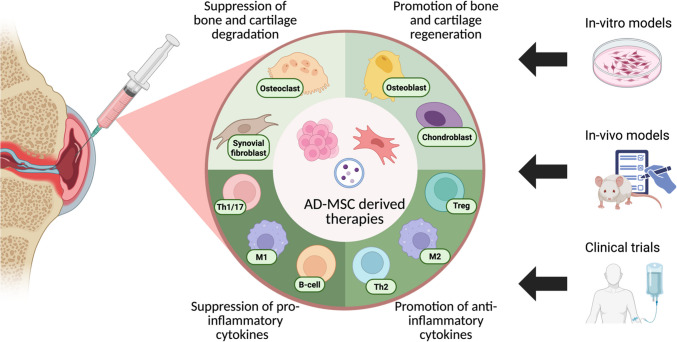

## Background

### Overview of Common Arthritis Types and Their Current Management Strategies

The synovial joint (SJ) facilitates the movement between adjacent bones ensuring a physiological range of motion. The joint cavity contains synovial fluid (SF), an ultrafiltrate of blood plasma that is secreted by the synovial membrane (synovium) which lines the articular capsule. The SF is primarily composed of hyaluronan, lubricin, proteinase, collagenases, and prostaglandins [1]. Hyaline cartilage forms the articular cartilage, which is continuous with the synovial membrane, covering the entire articulating surface of each bone [2]. SJs are comprised of a collection of tissue types enclosed within a sheath and, as such, is considered as being an organ. Like other organs, SJs are targets for various disorders, such as autoimmune diseases, infections, trauma, and, most commonly, degenerative changes such as those found in osteoarthritis (OA) [3]. Inflammatory joint diseases affect the entire synovial joint, with pathological changes observed in joint components, including osteoblasts, chondrocytes, synovial fibroblasts, synovial macrophages, adipocytes, tenocytes, ligament fibroblasts and endothelial cells [4], as well as the modified inflammatory milieu of immune cells [5]. SJ disorders, such as OA and rheumatoid arthritis (RA), afflict a substantial proportion of the global population, reportedly reaching 7% [6] and 1% [7] worldwide, respectively.

Arthritis is broadly classified into inflammatory and non-inflammatory types, both including a plethora of etiologies [8]. Inflammatory arthritis is comprised of RA, psoriatic arthritis (PsA), and peripheral spondyloarthritis (SpA) [9]. Additional arthritic conditions include mono-arthritis [10], and oligo-arthritis or polyarthritis, depending upon the number of affected joints. Gout and Pseudogout are inflammatory metabolic arthropathies caused by crystal deposits [11]. OA is the most prevalent form of arthritis and a leading cause of chronic pain and long-term disability in the elderly. The health and economic burden of OA is increasing in parallel with the ageing of the global population [12].

Inflammatory arthritis management has substantially improved patients’ quality of life and clinical outcomes [13]. The European League Against Rheumatism (EULAR) recommends starting treatment for newly diagnosed RA patients by means of conventional synthetic disease-modifying anti-rheumatic drugs (csDMARDs) therapy, mostly focusing upon methotrexate (MTX) [14], either alone or in combination with other csDMARDs or glucocorticoids (GCs). If the therapy is not well tolerated or ineffective, EULAR recommends using one of the following biologic DMARDs (bDMARDs): inhibitors of TNF, IL-6 receptor or CD20 antibody, a selective T cell co-stimulation modulator, CTLA-4 Ig, or targeted synthetic (ts) DMARDs (Janus kinase (JAK) inhibitors) [15]. For PsA patients, EULAR recommends treatment with csDMARDs (MTX) as an initial therapy. bDMARDs that include TNF, IL-17, IL-12/23, IL-23 inhibitors, or targeted therapies, such as phosphodiesterase-4, and JAK inhibitors are indicated if the treatment target had not been achieved [16]. GCs administrated through intra-articular (IA) injections are gold standard treatment for persistent joint inflammation [17].

There are no systemic approved disease-modifying drugs (DMOADs) for OA patients [18]. The most common therapeutic options to treat OA patients' joints are GCs administrated intra-articularly [19]. Joint replacement surgery is considered for severe cases of uncontrollable clinical disease with inadequate response to conservative treatment [13]. There are a few experimental alternative intra-articular (IA) treatment options, such as hyaluronic acid (HA) injections, platelet-rich plasma (PRP), and novel cell-based therapeutics [20]. Clinical outcomes with those novel experimental approaches, however, are often inconsistent, with different strengths recommended across guidelines [21].

### Mesenchymal Stem Cell (MSC) Therapies

MSCs are stromal cells that have self-renew abilities and exhibit multi-lineage differentiation. MSCs can be isolated from a variety of tissues, such as the umbilical cord, endometrial polyps, menses blood, bone marrow (BM), or adipose tissue [22]. MSCs derived from multiple sources have been used as cell-based therapy for decades due to their immunomodulatory and regenerative properties [23, 24]. Studies on MSCs for OA therapy have shown positive clinical outcomes, with improved joint function, pain level, and quality of life. Several clinical MSCs trials conducted on RA patients have also demonstrated some advantages [25, 26]. The largely positive outcomes without severe side effects in clinical trials have established MSCs as promising tools for arthritis therapy. However, further research is required to investigate its applicability in clinical settings [26]. This review will cover current *in-vitro* and *in-vivo* models in MSCs research for synovial joint disorder therapy, the results of clinical trials that investigated IA administration of MSCs, and the recent advancements in the potential application of cell-free therapy by means of MSCs-derived extracellular vesicles.

### Adipose Derived-Mesenchymal Stem Cells (AD-MSCs) in Regenerative Medicine

AD-MSCs have gained interest due to their therapeutic potential in regenerative medicine. Their low immunogenicity and their ability to self-renew, to differentiate into various tissue-specific progenitors, to migrate into damaged sites, and to act through autocrine and paracrine pathways have all been shown to be the main mechanisms by which cell repair and regeneration occur [27]. Unlike bone marrow-derived MSCs (BM-MSCs), AD-MSCs can be extracted in large, concentrated quantities (about 500 times more than BM-MSCs) by means of relatively simple procedures and under local anesthesia. Another benefit of AD-MSCs is that they can be extracted from various human body sites [26]. Furthermore, it has been shown that adipose tissue is superior to BM because of its safety and consistent efficacy in improving pain and functional outcomes [28].

## Effect of AD-MSCs on Synovial Cells- In Vitro Studies

**Fig. 1:**
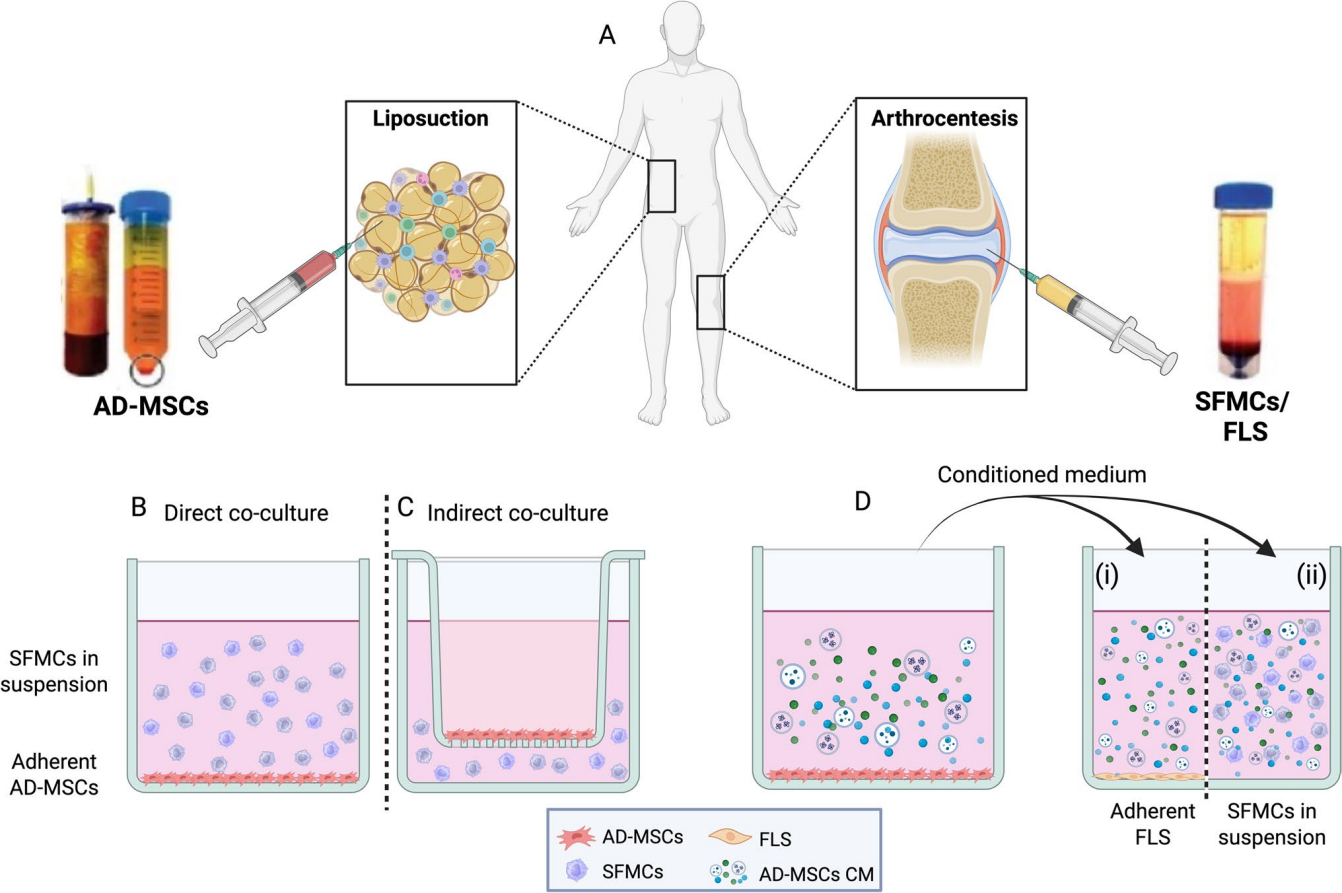
**A** Schematic illustration of collection and isolation of AD-MSCs and synovial cells. AD-MSCs are isolated from healthy donor white adipose tissue containing subcutaneous and internal (visceral and non-visceral) fat. SF are obtained by arthrocentesis from arthritis patients that exhibit persistent synovitis; synovial cells are then extracted by centrifugation from the SF specimen. **B** Direct co-culture model. The direct co-culture contains two different cell types. AD-MSCs are adherent monolayer cells while the SFMCs are grown in suspension. Cell–cell communication occurs through direct cell contact and can mimic those two cells types potential physiological interactions. **C** Indirect cell contact. Separation of two different cell types using a trans-well insert with a semipermeable membrane that allows communication via secretory factors. **D** Examination of the effect of transferred AD-MSCs conditioned medium (CM) on synovial cells. Secreted components in the AD-MSCs CM are transferred to (i) adherent fibroblast-like synoviocytes (FLS) or (ii) SFMCs in suspension

Most studies examining the effect of AD-MSCs on joint disorders rely upon *in-vitro* models in which synovial cells cultured with AD-MSCs are tested, providing information on their reciprocal effect as summarized in Table 1. These models enable the investigation of gene expression, proliferation, secreted proteins, cell populations, and cellular morphology. The different strategies to co-culture AD-MSCs with synovial cells are shown in Fig. 1. AD-MSCs are isolated from adipose tissue, while synovial cells are extracted from SF obtained via arthrocentesis from patients with synovitis (Fig. 1A). Direct and indirect co-cultures allow the investigation of different types of cellular communication, like juxtacrine (Fig. 1B) and paracrine (Fig. 1C) signaling, through the degree of exposure different cell types have on each other. In Fig. 1B and C shown are synovial fluid mononuclear cells (SFMCs) a cell fraction grown in suspension. Figure 1D shows examination of AD-MSCs conditioned medium (CM) on two synovial cell types: SFMCs (in suspension) and adherent fibroblast-like synoviocytes (FLS).

**Table 1:** Summary of in vitro findings of AD-MSCs in co-culture systems

Culture system	Cell source	Effect	Ref
Direct co-culture	Peripheral blood mononuclear cells (PBMCs) and AD-MSCs, previously primed with or without IL-1β	AD-MSCs modified CD4^+^/CD8^+^ T cell ratio	[29]
	AD-MSCs and activated-M1-like macrophages differentiated from PBMCs and digested OA synovium	AD-MSCs responsible for the switching of activated-M1-like inflammatory macrophages to a M2-like phenotype	[30]
	RA patients SF-primed AD-MSCs, CD4^+^ T cells and monocytes obtained from peripheral blood samples	The effect of SF on the ability of AD-MSCs to modulate the phenotype of macrophages or T cells was evaluated	[31]
	MSCs line with chondrocytes isolated from OA patients	3D co-culture induced suitable conditions for MSC differentiation toward chondrocytes better than 2D standard culture	[32]
Indirect–transwell co-culture	AD-MSCs in the upper chamber and peripheral blood monocytes (CD14^+^) in well	The ability of AD-MSCs to modulate the macrophage phenotype was enhanced after priming with IL-1β	[29]
	AD-MSCs in the upper chamber and activated-M1-like, M2-like macrophages and SF cells in the lower chamber	Migration assays were performed to determine if M1-like, M2-like macrophages, and SF cells influenced the migration of AD-MSCs. The effect of AD-MSCs on the activated-M1-like macrophage phenotype was also evaluated	[30]
	Primary chondrocytes in the lower chamber and AD-MSCs in the upper chamber. + Primary chondrocytes in the upper chamber and AD-MSCs in the lower chamber	The effect of AD-MSCs on articular chondrocyte proliferation and migration and the expression of markers of chondrocyte re-differentiation were evaluated	[33]
	AD-MSCs in the upper chamber and primary chondrocytes in the lower chamber	The paracrine effect of AD-MSCs on chondrocytes was evaluated	[34]
	CD11b^+^ cells were seeded in the upper chamber and AD-MSCs were plated in the lower chamber	The dependence of the AD-MSCs on osteoclastogenesis suppression was determined	[35]
Indirect transferred medium	TNFα-primed MSCs medium with human synovium or cartilage explants	Paracrine effects of MSCs on OA cartilage and synovial explants were shown	[36]
	Human articular chondrocytes from OA patients and CM from AD-MSCs	The ability of AD-MSCs-CM to regulate mediators involved in cartilage degeneration in OA chondrocytes was investigated	[37]

Giannasi et al. compared the secretome composition of CM collected after 24 h or 72 h from untreated MSCs and from cytokine-primed MSCs with either tumor necrosis factor α (TNFα) and/or interleukin 1β (IL-1β). The pre-activation of AD-MSCs aimed to mimic the pathological milieu of OA joints in order to target their secretion towards a specific pathological phenotype. Their results indicated that pre-conditioning AD-MSCs with inflammatory cytokines induced cell activation and led to the enrichment of their secretome by several factors with recognized anti-OA effect, including anti-inflammatory, chondroprotective, and analgesic properties [38]. These findings have also been confirmed by Colombini et al.’s group, who demonstrated an enhanced immunomodulatory potential of IL-1β-primed AD-MSCs on macrophages [29]. Another study examined the role of AD-MSCs in switching activated M1-like inflammatory macrophages into M2-like anti-inflammatory phenotype, partially through the PGE2/COX2 pathway, as the authors demonstrated with isolated OA synovial macrophages. This capability supports a specific role of AD-MSCs in the therapeutic resolution of OA synovial inflammation, as well as providing evidence that IFNγ-activated M1-like macrophages represent a good cell model capable of testing AD-MSCs activity [30].

Migration plays an important role in the therapeutic function of stem cells. AD-MSCs can migrate toward tissue injury sites in response to high levels of chemokines [39]. In order to assess such AD-MSCs activity, transwell inserts with large (8 μm) porous were used, allowing cells in the upper chamber to migrate through the pores. The degree of migration of different cells can then be quantified [e.g. chondrocytes [33], B [40] and T cells [41]] with or without AD-MSCs.

Zhang et al. showed that AD-MSCs significantly promoted the proliferation and migration of primary articular chondrocytes when co-cultured together, suggesting that AD-MSCs may act upon endogenous chondrocytes by secreting trophic factors. Co-cultured AD-MSCs also significantly upregulated the expression of articular chondrocyte re-differentiation markers and downregulated the expression of articular chondrocyte de-differentiation markers at the mRNA and protein levels, suggesting that AD-MSCs were capable of forming high-quality biomechanical articular cartilage *in-vivo* [33].

It is important to take into account the effects of synovial cells on AD-MSCs when considering AD-MSCs as a possible therapy for inflammatory synovial disorders. Sayegh et al. demonstrated that the immunomodulatory efficiency of AD-MSCs is highly dependent upon the cytokine/articular microenvironment. AD-MSCs cultured with pro-inflammatory SF of RA patients maintained their proliferation capability and upregulated gene expression involved in immunomodulation through a TNF/NF-κB-dependent mechanism. Their results also showed that AD-MSCs exposed to pro-inflammatory SF were more effective in inducing regulatory T cells (Tregs) and inhibiting pro-inflammatory macrophages compared to control SF obtained from a patient undergoing a trauma-related surgery [31]. Another group co-cultured SF from OA patients with AD-MSCs with the aim of mimicking the joint cavity environment. Their results showed an increase in AD-MSCs viability following SF supplementation as well as changes in several genes involved in cell survival. For example, FOS like 1 (FOSL1), which is involved in the therapeutic effect of AD-MSCs, was upregulated after SF exposure. Those authors suggested that future AD-MSC-based therapy may be further enhanced in an environment that facilitates upregulation of the expression of FOSL1, an AP-1 transcription factor subunit, expression [42]. Wesdorp et al. [43] studied the influence of synovial inflammation on MSCs migration, and whether modulation of inflammation with GCs (triamcinolone acetonide [TAA]) may influence migration. Those authors found that macrophages secrete factors that stimulate MSCs migration, and that modulation with TAA specifically increased the ability of anti-inflammatory macrophages to stimulate MSCs migration. They suggested that modulating inflammation and thereby improving MSCs migration could be a therapeutic approach based upon endogenous repair of cartilage defects.

It has been suggested that the 3-dimensional (3D) spheroid, which is a multicellular aggregate formed under non-adherent 3D *in-vitro* conditions, may be a useful *in-vitro* method to better understand MSCs therapy since expression of the extracellular matrix (ECM) and other adhesion proteins as well as immunomodulatory and anti-apoptotic genes are upregulated in that cell culture system [44]. AD-MSCs spheroids could therefore emerge as a promising OA treatment, although studying the appropriate methods of administration and considering their interaction with SF is essential. Toward this end, Fuku et al. [45] examined the effect of patient SF on 2D-culture AD-MSCs and spheroid AD-MSCs and observed that SF exhibited a lethal effect on AD-MSCs in 4 patients. This is, to the best of our knowledge, the first report of such an effect of SF on AD-MSCs. Although many positive effects have been attributed to the formation of AD-MSC spheroids, Fuku’s group emphasized that the usefulness of spheroids in OA treatment should be examined in experimental conditions that are more suitable for the joint cavity environment and in a larger number of cases. They recommended that the cytotoxic activity of SF against AD-MSCs administered for therapeutic purposes into the joint cavity should be considered when improving culture methods [45].

It has been shown that AD-MSCs secretum, also known as CM, possesses effects like those observed after transplantation of MSCs [46], containing many cytokines, proteins, growth factors, as well as extra cellular vehicles (EVs) with beneficial effects that can be used in regenerative medicine [47]. For example, one group treated synovium and cartilage explants with MSCs-CM to study the paracrine effect of MSCs secretome. Additionally, they used a co-culture system of synovium and cartilage explants, while preventing direct contact between cartilage and synovium, using filter inserts, creating a more complex and clinically relevant model to test AD-MSCs secretome. Using this culture setting, they found that synovial explants exposed to MSCs-CM expressed less pro-inflammatory factors, such as IL-1β, matrix metalloproteinase (MMP) and had reduced nitric oxide (NO) production, while upregulating multiple anti-inflammatory and anti-catabolic pathways in osteoarthritic cartilage and synovium [36].

### Potential Application of AD-MSCs-Derived Extra-Cellular Vesicles (EVs)—In Vitro Studies

**Fig. 2:**
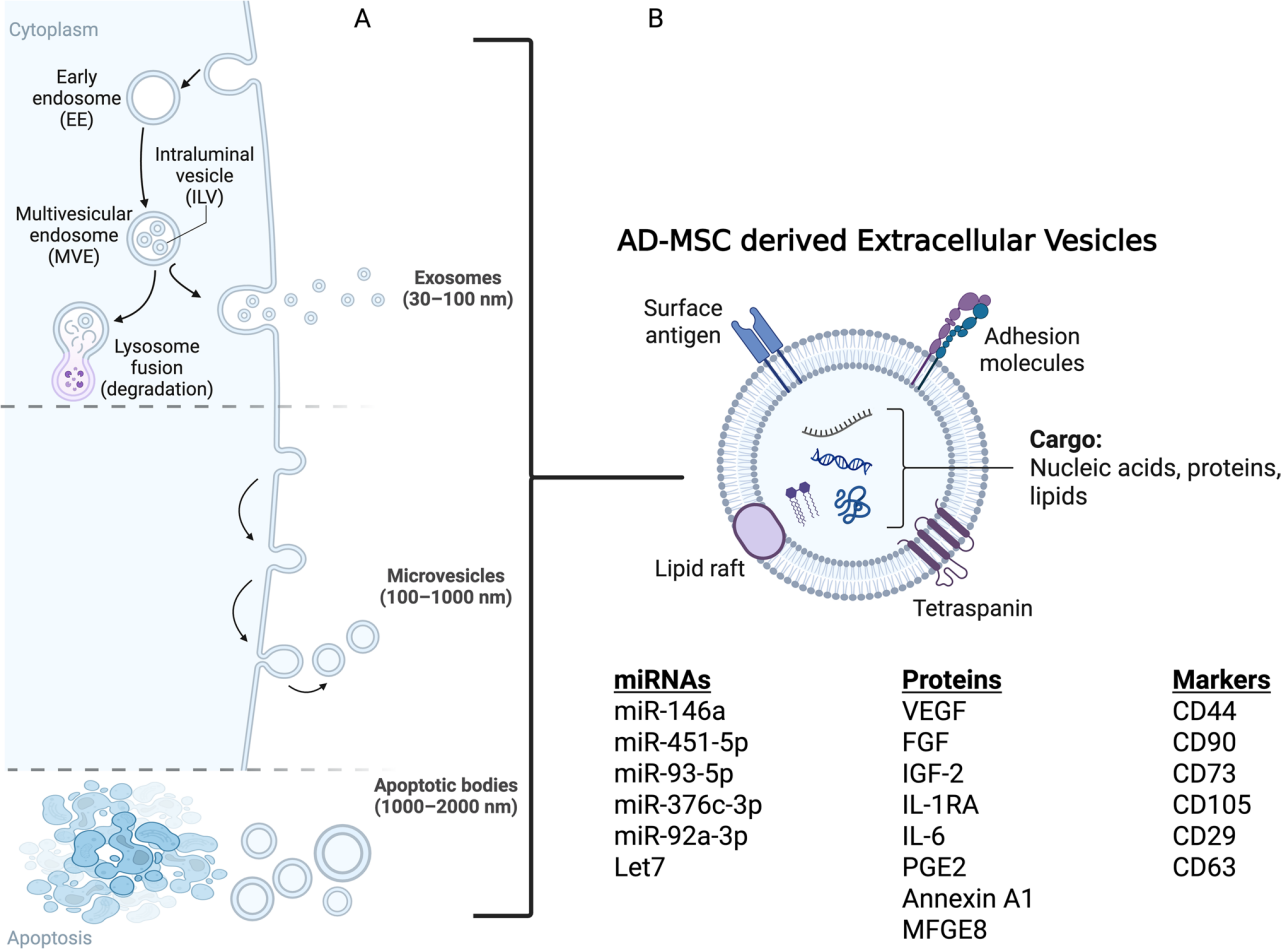
AD-MSCs-derived extra-cellular vesicles (EVs) **A** Types of EVs; exosomes (30–100 nm), microvesicles (100–1,000 nm), and apoptotic vesicles (1–5 mm) and their mode of secretion into the extracellular space. **B** Illustration of EVs encapsulation that contains biologically active cargos including proteins, nucleic acids, lipids and specific surface markers

EVs comprise a heterogeneous group of vesicles released by various cell types, EVs are released from cells but cannot replicate on their own. EVs can be classified into different subtypes based on their diameter: exosomes (30–100 nm), microvesicles (100–1,000 nm), and apoptotic vesicles (1–5 mm) [48] (Fig. 2A). Several studies have indicated that EVs can transfer their contents to elicit functional responses and mediate signaling through surface receptor contact between cells. EVs contain thousands of different bioactive molecules, including surface proteins, intercellular proteins, amino acids, metabolites, mRNAs, non-coding RNA species, and DNA [49] (Fig. 2B), playing an active role in various processes, such as angiogenesis, antigen presentation, cellular homoeostasis, inflammation, and immunomodulation. EVs derived from various types of MSCs have recently been explored as a novel strategy for cell-free therapy in a variety of diseases, including RA. Based upon current evidence, EV-derived MSCs have been demonstrated to be important MSCs mediators that exert similar functions, and that they can perform the indispensable functions of their parental cells. AD-MSC-derived EVs (AD-MSC-EVs) are thought to function similarly to AD-MSCs.

Although several studies have shown that AD-MSCs could ameliorate RA, the ones that explored the role of AD-MSC-EVs for RA treatment have emerged only recently [49].

Farinazzo et al. showed that AD-MSC-EVs can partially inhibit T cells activation *in-vitro*, but that this effect was insufficient *in-vivo*. In addition, AD-MSC-EVs were shown to drive M2 macrophage polarization, thus reducing the ability of macrophages to evoke inflammatory responses. Interestingly, Rossana et al. [50] demonstrated that only the EVs that had been isolated from AD-MSCs pre-activated with IFN-γ and TNF-α induced M2 macrophage polarization. Those authors therefore suggested that the effects of the immunomodulatory AD-MSCs-EVs on macrophages may not be constitutive but rather induced by the inflammatory microenvironment.

The therapeutic ability of MSCs to address OA mainly relates to the secretion of bioactive factors, which can be found within their secreted EVs. Cavallo et al. [51] demonstrated the potential of AD-MSC-derived small EVs (sEVs) by showing their effect on gene expression and protein release of both chondrocytes and synoviocytes by counteracting IL-1β-induced inflammatory effects. The inflammatory environment mediated by NF-κB pathway was significantly attenuated by sEVs. This approach can potentially be used as a new therapeutic strategy for OA therapy. Another group showed that EVs reduced the production of inflammatory mediators TNF-α, IL-6, PGE2, and NO in OA chondrocytes stimulated with IL-1β. Treatment also decreased the release of MMP activity and MMP-13 expression, whereas the production of the anti-inflammatory cytokine IL-10 and the expression of collagen II were significantly enhanced [52].

In a similar model, Tofiño-Vian et al. showed that EVs from AD-MSCs reduced inflammation and oxidative stress, mostly through IL-6 and prostaglandin E2, which may mediate anti-senescence effects in OA osteoblasts, seen by the downregulation of senescence-associated β-galactosidase activity and the reduced accumulation of γH2AX foci [53].

Among the bio-active molecules in AD-MSC-EVs microRNAs (miRNAs), e.g. miR-146a [54], miR-451a [55], miR-93-5p [56], miR-376c-3p [57], have been shown to directly alleviate local inflammation and promote tissue repair. Engineering AD-MSCs, whether by pre-activation or direct transfection, is used as a novel approach for functionalizing AD-MSC-EVs for a more potent immunomodulatory and regenerative effect. Li et al. showed that miR-338-3p overexpressing AD-MSCs exosomes could inhibit chondrocyte inflammation and degradation, as seen by decreased expression of PGE2, IL-6, IL-1β, TNF-α, MMP-3 and MMP-13, as well as by promoting chondrocyte proliferation through targeting RUNX2 [58]. Martinez-Zalbidea et al. transduced an immortalized AD-MSCs line with a CRISPR activation lentivirus system that overexpressed the TSG-6 gene and demonstrated their immunomodulatory potential in IL-1β activated intervertebral disc cells, specifically downregulating IL-8 and COX-2 [59].

Adipose tissue-derived autologous cells are easier to obtain for cell-free therapy in large-scale populations, making adipose tissue a richer and safer source compared to other tissues. Proteomic analyses have shown that AD-MSC-EVs are more closely associated with immunomodulation-related proteins than those from BM-MSC-EVs [60]. In addition, AD-MSC-EVs have been shown to be more effective than BM-MSC-EVs at promoting cartilage and bone regeneration in a mouse model and they represented a superior resource for cell-free therapy [61]. Woo et al. demonstrated that AD-MSC-EVs not only promoted the proliferation and migration of human OA chondrocytes, but that they also maintained the chondrocyte matrix by increasing type II collagen synthesis and decreasing MMP-1, MMP-3, MMP-13, and ADAMTS-5 expression [62].

## In-Vivo Model Findings of the Effects of AD-MSCs on Synovial Cells

*In-vivo* models are necessary for translation of novel treatments to human trials. Understanding the effects of AD-MSCs on synovial cells requires the selection of animal models of various arthritic conditions. Here, we discuss experimental models used to investigate the effects of AD-MSCs on inflammatory arthritis, OA, and metabolic arthritis.

### Experimental Models for Inflammatory RA

*In-vivo* experimental animal models of inflammatory RA are used extensively to investigate the pathogenic mechanisms governing inflammation-driven joint damage. Collagen-induced arthritis (CIA) is the RA animal model most commonly used in basic research and drug development studies since it resembles the human RA features of typical synovitis and bone erosion manifestations. BM-MSCs were shown to inhibit T cell proliferation in vitro but could not inhibit CIA disease severity [63]. In contrast, MSC-derived EVs ameliorated CIA manifestations via induction of immunomodulatory T lymphocytes [64]. Methylated bovine serum albumin (mBSA) antigen-induced arthritis (AIA) is another model, and it includes local immunization with a protein antigen (BSA, ovalbumin) followed by IA administration to the affected joints. In contrast to other RA models, AIA occurs only in the injected joints and the immune response is confined to the affected joint where histological features are similar to those of RA [65]. IA administration of MSCs in that model showed reduced inflammation and cartilage damage [66], and MSCs-CM reduced disease severity and immune responses [67].

Proteoglycan-induced arthritis (PGIA) is induced by repeated immunizations with cartilage PG aggrecan in adjuvant, resulting in polyarthritis and subsequent articular cartilage and bone erosion [65]. Swart et al. [68] explored the immunosuppressive effect and mechanism of action of MSCs administrated via intraperitoneal (IP) and IA administration in a PGIA model. MSCs treatment, both via IP and IA, suppressed PGIA. MSCs were largely retained for weeks in the injection site. Those authors showed that MSCs treatment induced a more regulatory phenotype with the production of IL-4 and IL-10, but also of IFN-γ, and a systemic decrease of pathogenic antibodies, thus demonstrating the potential value of MSCs treatment in resistant arthritis.

The K/BxN model and G6PI-induced arthritis are transgenic mice expressing a transgenic T-cell receptor (TcR). These mice develop spontaneous arthritis, which was shown to be B-cell dependent. The transfer of IgG antibodies from arthritic K/BxN mice to naïve mice induced transient arthritis in many different mouse strains. It has been shown that AD-MSCs therapy was unsuitable for modulating the progression of K/BxN serum-transfer arthritis. Based upon the differences in the immune status and monocytic/macrophage balance among the different arthritic models, it was suggested that the identification of cell-specific targets of MSCs will assist in predicting which RA patients will respond positively to MSCs-based therapy [69]. Wang et al. showed that IA administration of AD-MSCs attenuates RA progression by suppressing pro-inflammatory cytokines, and mitigating neutrophil infiltration and cartilage erosion, as well as promotes the reconstruction of the CX3CR1^+^ synovial macrophage barrier in serum-transfer arthritis model mice [70].

The SKG arthritis model was developed using a point mutation in the gene coding for ZAP-70, affecting TcR signaling and eventually leading to an effect upon thymic selection of T cells and, subsequently, autoimmunity. Local transplantation of AD-MSCs and AD-MSC spheroids had a significant therapeutic effect in that model [71]. Another group showed that the administration of AD-MSCs in the SKG arthritis model has a paradoxical effect according to the disease phase during which they were administered, suggesting that the cells act differently depending upon the disease stage [71].

Transgenic mice that overexpress human IL-1α are used to study the role of IL-1 in arthritis. These mice develop spontaneous polyarthritis, synovial proliferation, and cartilage and bone destruction. The cellular composition in joints changed in favor of polymorphonuclear neutrophils (PMNs) and macrophages [72]. Lee et al. [73] investigated the anti-inflammatory effect of BM-MSCs in an IL-1RaKO mouse model. Those authors demonstrated that BM-MSCs inhibited Th17 polarization and thereby reduced inflammation in those mice.

### Experimental OA Models

Current animal models for OA can be broadly classified into subtypes, as described by Samvelyan et al. [74], including naturally occurring, genetically modified, surgically induced, chemically induced, and non-invasive models. Spontaneous OA models, such as naturally occurring and genetically modified ones, are commonly used to study primary OA, while invasive models, such as surgically and chemically induced ones, as well as non-invasive models are used to investigate secondary OA. Invasive models are largely used to assess the efficacy of potential OA treatments. Li et al. [75] injected AD-MSCs to rats in an OA model induced by medial meniscectomy of the knee for 8 weeks prior to AD-MSCs injection. AD-MSCs transplantation was accompanied by enhanced cartilage regeneration in those rats. Their study also demonstrated the pharmacokinetics and pharmacodynamics of labeled AD-MSCs in the injured rat joints 10 weeks after their IA administration. The authors concluded that AD-MSCs persisted locally for 10 weeks in the rat joint, coinciding with the efficacy observed. It is postulated that persistence and/or proliferation of the AD-MSCs in the joint is required to exert their functions in promoting joint regeneration and/or cartilage protection, further supporting the safety and feasibility of IA administration of AD-MSCs for OA treatment [75].

Zhou et al. treated OA rats with IA administration of AD-MSCs, which alleviated OA and inhibited cartilage degeneration by suppressing cartilage apoptotic damage and reducing the pro-inflammatory cytokine secretion [76]. In contrast, repeated IA administration of allogeneic BM-MSCs resulted in an adverse clinical response and aggravation of the OA. Although there were no differences in clinical parameters between autologous and allogeneic MSC-treated equines with OA after the first IA administration of these cells, there was an adaptive immune response and aggravation of OA following a second exposure [77].

Mei et al. [78] evaluated the efficacy of IA administrations of culture-expanded allogenic AD-MSCs for the treatment of anterior cruciate ligament transection (ACLT)-induced rat OA. Their results supported the protective effect of AD-MSCs on cartilage degeneration, without any notable adverse effects. MHC-unmatched AD-MSCs protected chondrocytes from inflammatory factor-induced damage. Those results revealed that the paracrine effect of AD-MSCs on chondrocytes contributes, at least in part, to the therapeutic effect of AD-MSCs in OA. Similarly, Kuroda et al. [34] found that IA administrated AD-MSCs inhibited cartilage degeneration progression in an ACLT-OA rabbit model. That effect was mediated by AD-MSCs homing to the synovium and by the secretion of factors with chondroprotective effects, such as regulating chondrocyte viability and cartilage matrix protection, specifically thorough inhibiting MMP-13. Cheng et al. used combined IA AD-MSCs and shockwave (SW) therapy in an ACLT OA rat model. The combination therapy had a chondroprotective effect as evidenced by a significantly increased bone volume, trabecular thickness, and trabecular number, as well as a significantly reduced synovitis score compared to the other treatments [79].

The use of uncultured adipose-derived stromal vascular fraction (SVF) consisting of AD-MSCs as well as M2 macrophages has shown therapeutic potential for OA. Onoi et al. induced OA in immunodeficient rats by destabilization of the medial meniscus and immediately injecting human SVF, AD-MSCs, or saline (control group) into the knees. The SVF group showed significantly slower OA progression and less synovitis, with higher expression of collagen II and SOX9, lower expression of MMP-13 and ADAMTS-5, and a lower M1/M2 ratio in the synovium compared with the AD-MSCs and control groups [80]. Ko et al. [81] used another therapeutic approach consisting of 3D-cultured AD-MSCs. Those authors evaluated the effect of spheroidal AD-MSCs administration on cell survival and arrest the progression of surgically induced OA in a rat model. Their results showed that AD-MSCs spheroids had better *in-vitro* and *in-vivo* survival and better chondrogenic potential, and that they exerted greater regenerative effects than AD-MSCs in single-cell suspension, probably by a paracrine mode of action. These findings support the use of stem cell-based therapeutics upon AD-MSCs spheroid forms to treat OA. Similarly, Yoon et al. [82] compared the chondrogenic differentiation capabilities of AD-MSCs in monolayer and culture in 3D suspension bioreactors. Enhanced in vivo cartilage formation was achieved by the transplantation of spheroid-cultured AD-MSCs compared to monolayer-cultured AD-MSCs. The authors attributed this to increased TGF-β3 expression and p38 activation. Their results showed that spheroid culture may be an effective method for large-scale *in-vitro* chondrogenic differentiation of AD-MSCs and subsequent *in-vivo* cartilage formation.

Pain is a recurrent symptom in OA and the primary reason for medical intervention. Amodeo et al. [83] explored the efficacy of AD-MSC-CM in controlling pain and neuroinflammation in OA. Their monosodium-iodoacetate (MIA)-induced OA mouse model is largely used to evaluate pain resulting in functional impairment similar to human OA. Those authors demonstrated that AD-MSC-CM exerts a rapid and lasting relief of pain symptoms, probably through modulation of neuroinflammation. They also reported that systemic injection of AD-MSC-CM appears to be the most active route.

### Experimental Models for Metabolic Arthritis

Several animal models of hyperuricemia and gouty arthritis have been reported for *in-vivo* assessment. The injection of monosodium urate (MSU) crystals into various anatomical structures to induce crystal-induced inflammation has been proposed; however, only a few of these models accurately reflect the joint microenvironment in which an acute gouty attack occurs [84]. The pathogenesis of gouty arthritis, characterized by the MSU crystal deposition in the joints associated with acute flares, has been associated with NOD-, LRR-, and pyrin domain-containing protein 3 (NLRP3) inflammasome activation and subsequent amplification of the inflammatory response. Medina et al.’s study tested the effect of AD-MSCs administration on the clinical inflammatory response in rabbits after MSU injection. A single dose of AD-MSCs was injected IA shortly after IA administration of MSU crystal. There was both local and systemic inflammatory response resolution in this model. Furthermore, AD-MSCs promoted M2 macrophages in the synovial membrane, inhibited NLRP3 inflammasome, and induced anti-inflammatory cytokines, IL-10 or TGF-β, while decreasing NF-κB activity [85]. The few *in-vivo* models that have thus far been described in the literature for PsA include three main categories of animal models for PsA: spontaneous/gene mutant types, transgenic types, and induced types [86]. MSCs were not employed for treatment of PsA in those models.

### Potential Application of AD-MSCs-Derived Extra-Cellular Vesicle (EVs)—In Vivo Studies

In animal models, Woo et al. showed that IA administration of AD-MSC-EVs significantly attenuated OA progression and protected cartilage from degeneration in two OA models [62]. In addition, MSCs exosomes were shown to reduce pain and repair temporomandibular joint osteoarthritis (OA-TMJ) by mounting a well-coordinated response of attenuating inflammation, enhancing proliferation and matrix synthesis, while reducing apoptosis and matrix degradation to achieve overall joint homeostasis and promote TMJ repair and regeneration in an OA-TMJ animal model [87]. Tsujimaru et al. showed that AD-MSC-EVs possess immunosuppressive factors in their vesicles similar to AD-MSCs. They also showed that AD-MSCs and their derived EVs carry IL-1ra that effectively replaces its deficiency in an RA mice model, thereby improving their severe state. This effect has been shown to be comparable to that of anakinra, a recombinant IL-1ra-approved drug for RA management [88]. Wang et al. demonstrated the efficacy of exosomes derived from miR-486-5p overexpressing AD-MSCs in medial menisco-tibial ligament OA rat model. IA administration of miR-486-5p containing exosomes showed a better effect on cartilage preservation (decreased apoptosis, increased Col II and decreased Col X deposition), as well as synovial macrophage preservation (decreasing iNOS and increasing CD163 staining in synovium samples) [89].

## Clinical Trials Employing AD-MSCs for Arthritis Therapy

Clinical trials for evaluating the effectiveness of AD-MSCs for arthritis therapy in OA have shown promising results. Kim et al. conducted a clinical trial on 49 OA patients (55 knees) with symptoms of knee joint pain and/or functional limitations despite non-surgical treatments for a minimum of 3 months, AD-MSCs were harvested and loaded into a fibrin glue product and then surgically implanted into the lesion site. Clinical assessment scores showed significant improvement among the patients, with 74.5% of them expressing satisfaction with the resultant pain relief [90]. Interestingly, patients over 60 years of age who had lesion sizes > 6.0 cm^2^ showed less favorable results. Although there were some variations in the results due to these 2 factors, the overall clinical outcomes of MSC implantation in OA patients were encouraging.

Pers et al. [91] reported their phase I, prospective, bicentric, single-arm, open-label, dose-escalating clinical trial of AD-MSCs therapy in 18 OA patients who were injected IA. Despite the limited number of patients and the absence of a placebo arm, the authors were able to demonstrate the safety and promising treatment potential of AD-MSCs. Freitag et al. [92] conducted a randomized trial on 30 OA patients in whom AD-MSCs were extracted from their adipose tissues and cultured until passage 2 prior to injection. Autologous AD-MSCs were then IA administered in a single injection (group 1) or in 2 injections (group 2), while the control patients received conservative management (group 3). The AD-MSCs-treated groups showed clinically significant improvement in pain reduction and function. Magnetic resonance imaging analyses showed that 37% of the participants in the first treatment group exhibited further cartilage loss compared to the control. However, ~ 89% of the patients in the second treatment group showed marked improvement or no progression in cartilage loss. Furthermore, no serious adverse events were observed in the 2 treated groups during follow-up. The authors concluded that AD-MSCs therapy is a safe and effective treatment for OA and that frequent injections are preferable.

Lee et al. conducted a randomized, double-blinded, placebo-controlled study in 24 patients with knee OA aged 18–75 years who had a mean pain intensity (VAS score) of 4 or higher for a minimum of 12 weeks with at least 1 grade 3–4 lesion. The patients were randomly divided into those treated with IA AD-MSCs administration and a control group administrated with saline. The AD-MSC-treated group showed significant reductions in knee pain. Their physical and radiological examination data showed that they demonstrated a wider range of knee motion and unchanged cartilage defects, in contrast to the enlargement of defects seen in the control group. It was concluded that IA administrations of autologous AD-MSCs provided satisfactory functional improvement and pain relief for OA patients, and did so without causing short-term adverse events [26, 93]. In another study evaluating the safety and therapeutic potential of autologous AD-MSCs in OA patients, 18 OA patients were enrolled and divided into three dose groups. Clinical and radiological parameters were evaluated during 96 weeks of follow-up. The high-dose AD-MSCs group exhibited better pain relief and greater improvement in knee function compared to the other 2 groups [94].

Several studies have indicated that the inhibitory effect of AD-MSCs is affected by the stage of OA at which they are administered, favoring early-stage intervention with little to no effect in the late stage of the disease [95–97]. In addition, swelling of the injected joints is frequently observed and is thought to be associated with the survival rate of the AD-MSCs. Directly injected cells usually have limited cell retention and survival rates, especially in large cartilage lesions. Koh et al. reported that AD-MSCs seeded in scaffolds may have better viability, preservation, and aggregation. Those authors recommended that appropriate cell-loaded scaffolds should be developed for treating patients with large cartilage defects in order to improve the efficacy of this procedure, as well as the level of comfort of the patients [97].

Compared to OA, relatively few trials have been performed with MSCs in RA. In some cases, MSCs therapy may not be suitable for RA, given the growing armamentarium of other efficient therapeutics available as opposed to OA. Although there have been a limited number of clinical trials of MSCs, especially AD-MSCs for RA therapy, the therapeutic safety and efficacy have been confirmed in several studies [26]. Vij et al. conducted a phase I/IIa non-randomized, open-label study to evaluate the safety and efficacy of a single, intravenous infusion of autologous AD-MSCs over a period of 52 weeks in RA patients. The results indicated that it was safe and efficacious for the improvement of joint function in those patients [98]. In a multicenter, dose escalation, randomized, single-blind, placebo-controlled phase Ib/IIa clinical trial of 53 patients, Álvaro-Gracia et al. demonstrated that intravenous infusion of culture-expanded AD-MSCs was generally well tolerated and free of side effects at the dose range and time frame studied [99]. In their double-blind, ascending dose, Phase I/IIa randomized controlled trial, Freitag et al. sought to assess the safety and efficacy of an allogeneic AD-MSCs preparation (MAG200) in the treatment of knee OA over 12 months. Their results showed a reproducible and statistically significant effect over placebo, which was clinically relevant for pain in the 10 ​ × ​10^6^ dose cohort and for function in the 20 ​ × ​10^6^ and 100 ​ × ​10^6^ dose cohorts. Furthermore, a trend in disease-modification was observed, with improvement in total knee cartilage volume in MAG200 10, 20, and 100 ​ × ​10^6^ dose cohorts, but with progression of the OA in placebo group, though this was not statistically significant [100].

**Fig. 3:**
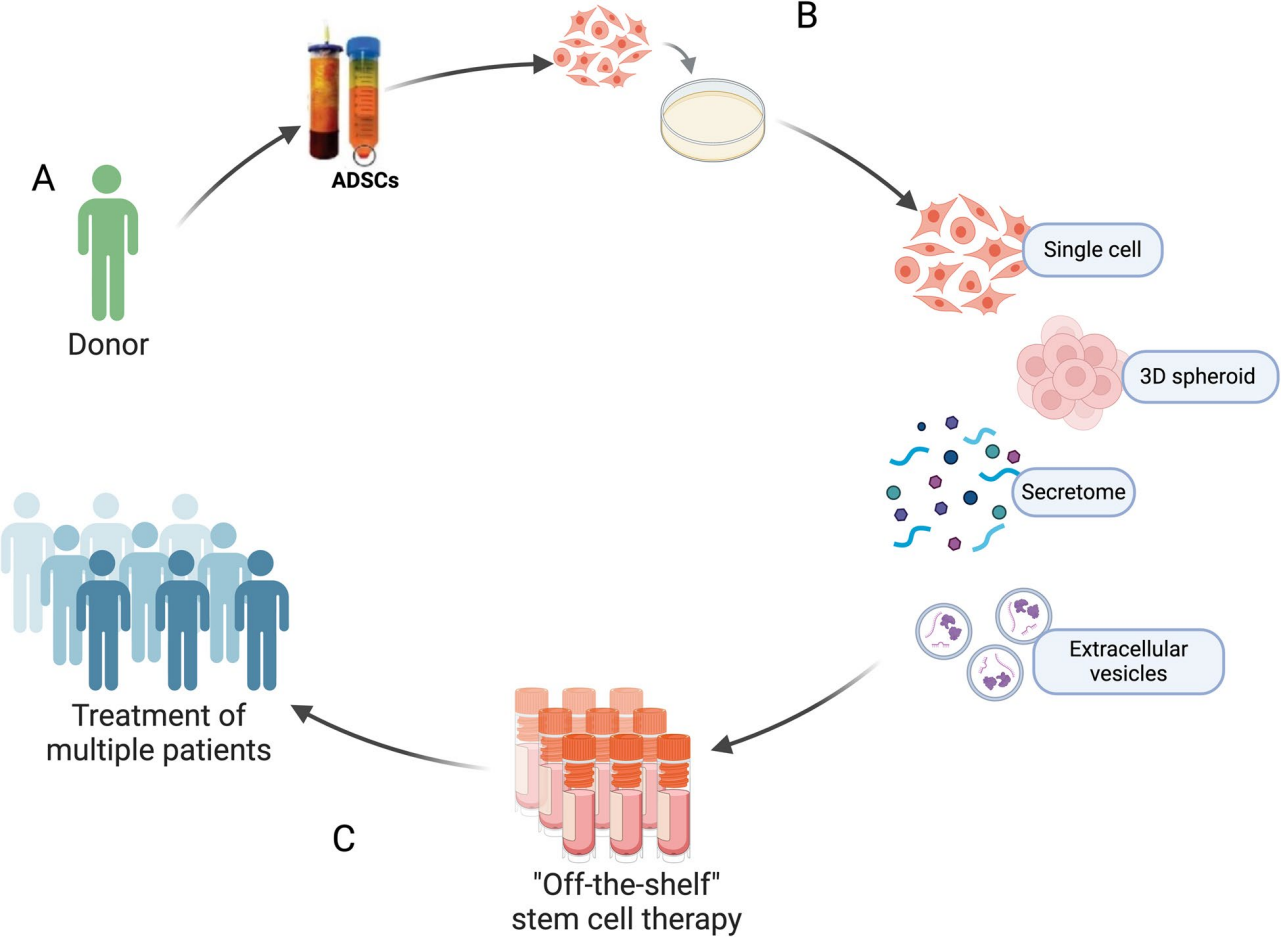
Standardization of AD-MSCs and AD-MSC-derived therapies may promote the translation into clinical practice **A** Identification of suitable donor candidates for AD-MSCs source isolation, being syngeneic or allogeneic transplantation, maximizing treatment potency. **B** Production and processing of a variety of AD-MSCs and AD-MSC-derived therapies insuring both quality and safety. **C** Stem cell-based therapy can be translated to clinical practice and potentially become an off-the-shelf treatment for the wide population

Chen et al. evaluated the safety and efficacy of IA administration of allogeneic AD-MSCs (ELIXCYTE®) for OA knee among 57 subjects in a patient-blind, randomized, active-control trial consisting of 4 arms that consisted of a hyaluronic acid control and 3 ELIXCYTE® doses. The results showed a significantly reduced pain score at week 24 between the post-treatment visit and baseline in all ELIXCYTE® groups. Compared to HA group, those groups revealed significant decreases at week 4 in total pain scores, stiffness scores, and functional limitation scores, suggesting the potential benefit of ELIXCYTE® in earlier onset cases [101]. Similarly, Kuah et al. investigated Progenza (PRG) *in-vitro* expanded AD-MSCs with cell culture supernatant as potential disease-modifying treatment options for OA. Their single center, randomized, double-blind, placebo-controlled, single ascending dose study showed significant within-group improvement in pain scores from baseline at all-time points for the PRG group, with highly significant improvements seen at months 3, 6, 9, and 12 compared to the marginal improvement in the placebo group [102]. Although its safety has been extensively demonstrated, it is important to note that allogenic AD-MSCs IA administrations might have serious consequences in certain conditions. For example, Hosono et al. reported the development of severe arthritis caused by multiple IA administration with AD-MSCs for knee OA. A total of 113 patients received AD-MSCs treatment, and 75% (85/113) of them received an AD-MSCs injection at least twice with a 6-month interval. Although no obvious negative sequelae were observed in any patient after the first treatment, 53% (45/85) of the patients who received their second or third AD-MSCs injections sustained severe knee arthritis. In terms of pathophysiology, Hosono et al. found anti-histone H2B Ab in SF samples from patients who exhibited severe arthritis following the second AD-MSCs treatment [103]. Although more research is needed to determine the duration of the therapeutic effects, the optimal dosing, and the administration strategy, as well as recipient patient profiles for treatment candidates, AD-MSCs are considered to be low-risk and suitable for treatment. The source of AD-MSCs, whether autologous (derived from the patient) [90–95, 98] or allogenic (donor-derived) [99–104], as well as additional donor characteristics (Fig. 3A), should also be addressed in future research and therapeutic decisions.

Finally, MSCs and their derived products have immunomodulatory, chondro- and osteo-protective potential via functional changes of T cells, monocytes/macrophages, and synovial cells that help restore the synovial hemostasis, as shown in the graphical abstract. Many pathways relating to the pathogenesis of arthritis have been shown to be affected by AD-MSCs. Table 2. summarizes the underlying mechanisms of AD-MSC-mediated immunomodulation and tissue regenerative abilities across experimental models. AD-MSCs treatment has consistently been shown to reduce the levels of IL-1β and TNFα, key cytokines involved in the inflammatory response and the development of structural bone and cartilage damage in arthritis. In contrast, IL-10 and TGF-β, key regulators of immune homeostasis, are consistently increased by AD-MSCs treatment. These findings coincide with phenotypic changes from inflammatory T-cells to Tregs and from M1-macrophages to M2-macrophages.

**Table 2:** Summary of the effects of AD-MSC effects on disease-associated inflammatory cells/cytokines across arthritis models

	AD-MSCs treatment type	Model	Results	Reference
	T-cells			
In-vitro	AD-MSCs CM	RA human PBMCs	↑ TGF-β, ↑ Tregs, ↓ Th17, ↓ TNFα, ↓ IL-17, ↓ IL-21	[105]
	Allogeneic AD-MSCs	Collagen-reactive RA human T cells; direct and transwell co-cultures	↓ IFNγ, ↓ TNFα, ↓ IL-17, ↑ IL-10, ↑ Tregs	[106]
	SF primed AD-MSCs	Activated CD4^+^ T cells; direct co-culture	↑ Tregs, ↓ Th1	[31]
	AD-MSCs	CD3^+^ T cells from CIA mouse lymph node	↓ IFNγ, ↓ TNFα, ↓ IL-2, ↑ IL-10, ↑ TGF-β	[107]
	AD-MSCs	Con A-stimulated murine splenocytes and human PBMCs; direct co-culture	↓ T-cell proliferation	[108]
In-vivo	Human AD-MSCs, allogeneic and syngeneic murine AD-MSCs	CIA mice; intraperitoneal injection	↓ Sera IgG, ↓ RANTES, ↓ Th1/Th17, ↓ IFNγ, ↓ TNFα, ↑ IL-10, ↑ Tregs	[107]
	AD-MSCs	CIA mice; Intravenous injection	↓ GM-CSF^+^CD4^+^ T cells (in spleen and peripheral blood but not in the draining lymph nodes), ↑ Tregs, Tr1, and LAG-3^+^Tr1 cells (in the draining lymph nodes)	[109]
	AD-MSCs	SKG mice; intraperitoneal injection	Con-A stimulated splenocytes; ↓ IL-17, ↓ IL-6, ↑ IL-10, ↑ TGF-β	[108]
Clinical trial	AD-MSCs	Phase Ib/IIa clinical trial	Distribution of T-cell populations, including Tregs in circulation, was not significantly modified among cohorts	[99]
	Monocytes/Macrophages			
In vitro	IL-1β primed Allogeneic AD-MSCs	Collagen-reactive RA human monocytes/macrophages; direct and transwell co-cultures	↑ IL-10	[106]
	IL-1β primed AD-MSCs	LPS\IFN-γ activated CD14^+^ monocytes from healthy donors; transwell co-culture	↑ CD206, ↑ M2-macrophages	[29]
	AD-MSCs	IFN-γ activated CD14^+^ monocytes from healthy donors; transwell co-culture	↓ M1-macrophages (↓ IL-1β, ↓ TNFα, ↓ IL-6, ↓ MIP1α/CCL3, ↓ S100A8, ↓ S100A9), ↑ M2-macrophages (↑ IL-10, ↑ CD163, ↑ CD206, ↑ PGE2	[30]
	SF primed AD-MSCs	LPS\IFN-γ activated CD14 + monocytes; direct co-culture	↓ CD40, ↓ CD80	[31]
	AD-MSCs and AD-MSC spheroids	PMA-differentiated, LPS activated THP-1 cells; co-culture	↓ TNFα, ↓ IL-6	[71]
	Murine inguinal fat AD-MSCs	CX3CR1^+^ synovial lining macrophages from healthy mice; direct and transwell co-cultures	Transcriptome related to leukocyte cell–cell adhesion, migration, and regulation of immune effector were significantly enriched and upregulated, e.g. M2 polarization; ↑ Rac2, ↑ CCL2, enhanced barrier function in CX3CR1^+^ macrophages; ↑ Tjp1, ↑ F11r	[70]
In-vivo	AD-MSCs and AD-MSC spheroids	SKG mice; IA administration	↓ Macrophage infiltration	[71]
	Murine inguinal fat AD-MSCs	K/BxN serum-transfer arthritis mice; IA administration	Synovium; ↓ IL-1β, ↓ TNFα, ↑ CX3CR1^+^ macrophages Peripheral blood; ↑ IL-10, ↓ IL-6, ↓ IL-1β	[70]
	Synovial cells			
In vitro	Allogeneic AD-MSCs	LPS\TNFα stimulated RA human FLS; direct and transwell co-cultures	↓ TNFα, ↓ PGE2, ↓ collagenase activity, ↓ gelatinase activity	[106]
	AD-MSCs	Collagen-reactive CIA mice synovial cells; direct and transwell co-cultures	↓ IL-1β, ↓ TNFα, ↓ MIP2, ↓ INFγ, ↓ IL-12, ↓ RANTES, ↑ IL-10, ↓ IL-17, ↓ IL-2, ↑ TGFβ	[107]
	AD-MSCs from infrapatellar Hoffa fat, SC hip fat, and SC abdominal fat	chondrocytes and synoviocytes; transwell co-cultures	↓ IL-1β, ↓ IL-6, ↓ CXCL8/IL-8, ↓ COX2, ↑ PGE2	[110]
	AD-MSC-EVs and CM	IL-1β activated OA chondrocytes and cartilage explants	↓ TNFα, ↓ IL-6, ↓ PGE2, ↓ NO, ↓ COX2, ↓ MMP activity, ↓ MMP-13, ↑ IL-10, ↑ collagen II	[52]
	AD-MSC-EVs and CM	IL-1β activated OA Osteoblasts	↓ TNFα, ↓ IL-6, ↓ PGE2, ↑ IL-10, ↓ mitochondrial oxidative stress, ↓ accumulation of γH2AX foci	[53]
	AD-MSC and AD-MSC spheroids	RA synovial fibroblasts; transwell co-cultures	↓ Migration, ↓ proliferation	[71]
	infrapatellar fat pad AD-MSC	IL‐1β activated OA articular cartilage cells	↑ COL2A1, ↑ Aggrecan, ↑ SOX9, ↓ MMP‐13, ↓ TNFα, ↓ IL-6, ↓ IL-1β, ↓ ROS	[111]
In-vivo	infrapatellar fat pad AD-MSC	Sprague–Dawley rats OA; IA administration	In synovial fluid; ↓ ROS, ↓ IL-6, ↓ TNFα, ↓ IL-1β	[111]
	Autologous AD-MSC and SVF	Sheep transected meniscus; IA administration	Condyle Immunohistochemistry; ↑ COLL2, ↓ COLL1, ↓ IL-1β, ↓ MMP13 Synovial Fluid; ↓ IL-1β, ↓ CTX2, ↓ TNFα, ↓ IL-6, ↓ PGE2	[112]
	AD-MSC derived exosomes	MIA-induced rat OA model; IA administration	cartilage tissues and rat synovial tissues; ↓ β-catenin, ↓ WNT3, ↓ WNT9, ↑ COL2	[57]

Also of note is the wide range of AD-MSCs treatment types, including different adipose tissue sources, culture methods, priming and processing approaches (Table 2), demonstrating both the need for further research for optimizing the therapeutic effect of AD-MSCs, as well as the need for standardization prior to clinical translation (Fig. 3B).

## Conclusions

AD-MSCs therapy for inflammatory and non-inflammatory joint disorders generally exhibited positive outcomes across research platforms, including *in-vitro* and *in-vivo* animal studies as well as in human clinical trials. However, despite its immense potential, only a few MSC-based therapies have been approved. It is vital for research groups to choose the appropriate models, both *in-vitro* and *in-vivo*, understand their limitations, and work to maximize their translatability. While significant progress has been made, additional research is needed to optimize AD-MSCs and AD-MSC-derived EV treatment strategies for joint disorders. Figure 3 shows the potential of future studies investigating AD-MSCs and their derivatives products towards clinical therapeutic use. The research needs to include platforms for evaluating the safety, optimal dosing, administration routes as well as profiles of patients who are candidates for such treatment (Fig. 3A). Standardizing the method of culturing, isolating and processing AD-MSC-derived treatments (Fig. 3B), could lead to their use in clinical application for standard arthritis care (Fig. 3C).

Additionally, further insights into the immunomodulatory mechanisms of AD-MSCs and AD-MSC-derived EVs in synovial joint disorders are necessary for successful translation into clinical practice. Overall, while AD-MSC-based therapies show promise for the treatment of joint disorders, further research is essential to realize their full therapeutic potential.

## Data Availability

All data used in this paper has been acquired from public domain and is available from the corresponding author upon request.

## Abbreviations

3D: 3-Dimensional
AIA: Antigen-Induced Arthritis
ACLT: Anterior Cruciate Ligament Transection
AD-MSCs: Adipose-derived mesenchymal stem cells
BM: Bone Marrow
bDMARDs: Biologic Disease-Modifying Anti-rheumatic Drugs
CIA: Collagen-Induced Arthritis
CM: Conditioned Medium
csDMARDs: Conventional Synthetic Disease-Modifying Anti-rheumatic Drugs
DMOADs: Disease-Modifying Drugs
ECM: Extracellular Matrix
EULAR: European League Against Rheumatism
EVs: Extracellular Vehicles
FLS: Fibroblast-like synoviocytes
FOSL1: FOS Like 1
GCs: Glucocorticoids
HA: Hyaluronic Acid
IA: Intra-articular
IL: Interleukin
IP: Intraperitoneal
MIA: Monosodium-Iodoacetate
MMP: Matrix Metallopeptidase
MSC: Mesenchymal Stem Cell
MSU: Monosodium Urate
MTX: Methotrexate
NLRP3: NLR family pyrin domain containing 3
NO: Nitric Oxide
OA: Osteoarthritis
OA-TMJ: Temporomandibular Joint Osteoarthritis
PBMCs: Peripheral Blood Mononuclear Cells
PGIA: Proteoglycan-Induced Arthritis
PMNs: Polymorphonuclear Neutrophils
PRP: Platelet-Rich Plasma
PsA: Psoriatic arthritis
RA: Rheumatoid arthritis
SF: Synovial fluid
SJ: Synovial joint
SpA: Spondyloarthritis
SW: Shockwave
TcR: T-cell Receptor
Tregs: Regulatory T cells
TNFα: Tumor Necrosis Factor α
